# Elevated Lipid Concentrations in Seminal Plasma Can Reduce Sperm Motility in Simmental Bulls

**DOI:** 10.3390/ani15020276

**Published:** 2025-01-20

**Authors:** Zhuo Yang, Fang Luo, Chenglei Song, Zhiyuan Ma, Yucheng Tian, Yu Fu, Hao Zheng, Jinzhong Tao

**Affiliations:** 1 College of Animal Science and Technology, Ningxia University, Yinchuan 750021, China; jennyyangzhuo@126.com (Z.Y.); luofang1001@163.com (F.L.); songchenglei99@163.com (C.S.); fuyu771202@163.com (Y.F.); 13213619969@163.com (H.Z.); 2 Station of Domestic Animal Improving and Management of Gansu Province, Wuwei 733000, China; mazy@163.com; 3 Ningxia Breeding Cow Biotechnology Co., Ltd., Zhongwei 751800, China; 13389551996@163.com

**Keywords:** CASA, sperm parameters, metabolic pathways, lipid profiles, biomarker

## Abstract

Sperm motility is a key factor influencing male fertility and is associated with metabolic and lipid profiles across species. The aim of this study was to investigate the relationship between sperm motility and the seminal plasma lipid profile in Simmental bulls, and to identify key lipids potentially influencing sperm motility. Semen samples were collected from 26 healthy Simmental bulls with an average age of 4.9 years. Sperm quality was evaluated using computer-assisted sperm analysis (CASA). Based on motility, the samples were divided into two groups: high sperm motility (HSM > 65%) and low sperm motility (LSM < 65%). Compared to the LSM group, the HSM group exhibited significantly higher sperm viability, motility, straight-line velocity, beat-cross frequency, and sperm acrosome integrity, while the sperm malformation rate was lower (*p* < 0.05). Lipid profiles were determined using LC-MS/MS, and 40 differential lipids were identified by multivariate statistical analysis. Among them, 39 lipids were upregulated in the LSM group compared to the HSM group. They were primarily triglycerides and carnitines, mainly involved in four metabolic pathways related to glycerophospholipid and linoleic acid metabolism. Notably, PC (16:0/20:4; 14:0/18:3), LPC (22:4/0:0; 22:6/0:0), and PE (14:0/18:1; 18:1/20:3) were diagnosed with great accuracy (AUC > 0.7), which means they may serve as potential biomarkers for sperm motility.

## Simple Summary

The aim of this study was to investigate the relationship between sperm motility and the seminal plasma lipid profile in Simmental bulls, and to identify key lipids potentially influencing sperm motility. The findings revealed that high-motility sperm exhibited higher straight-line velocity (VSL), beat-cross frequency (BCF), and acro-some integrity, as well as a lower sperm malformation rate compared to low-motility sperm. Additionally, the elevation of 39 differential lipids in seminal plasma, primarily enriched in glycerophospholipid and linoleic acid metabolism pathways, was associated with reduced sperm motility. Among these lipids, phosphatidylcholine (PC) (16:0/20:4), PC (14:0/18:3), lysophosphatidylcholine (LPC) (22:4/0:0), LPC (22:6/0:0), phosphatidylethanolamine (PE) (14:0/18:1), and PE (18:1/20:3) appeared to be applicable as biomarkers of sperm motility.

## 1. Introduction

Sperm motility directly impacts fertility rates and embryo quality and is influenced by several environmental and genetic factors [1,2]. Research has confirmed a close relationship between seminal plasma and the regulation of sperm motility. Seminal plasma is mainly composed of proteins, nucleosides, lipids, vitamins, amino acids, and steroid hormones [3]. Several studies have reported the effects of these components on sperm quality across various species. For example, in bulls, Therien et al. identified a specific correlation between apolipoprotein A-1 in seminal plasma and the regulation of sperm capacitation [4]. Hansen et al. reported a negative relationship between seminal plasma clusterin levels and the number of normal sperm in beef cattle [5]. In humans, Gilany et al. and Zhao et al. found metabolomic alterations in the Krebs cycle and energy metabolism pathways in the seminal plasma of patients with asthma-oligozoospermia [6,7]. In goats, Jia et al. demonstrated that 445 seminal plasma-derived proteins were significantly differed between high and low sperm motility groups [1]. Additionally, potential fertility biomarkers in seminal plasma have been identified, such as oleic acid and palmitic acid in humans [3], taurine, leucine, and isoleucine in bulls [8], and ethanolamine in stallions [9]. These findings highlight the complexity of the relationship between seminal plasma components and sperm motility, underscoring the need for further exploration in this area.

Lipids are major components of sperm membranes and are involved in sperm motility, energy metabolism, membrane function, and fertilization [10,11]. Around 24% of the sperm proteome in both humans [12] and horses [13] is devoted to lipid metabolism. Several studies have explored the effects of lipids in seminal plasma on sperm quality. For example, Kathein et al. found that lysophosphatidylcholines, phosphatidylcholines, and sphingomyelins in the seminal plasma of healthy donors are involved in regulating sperm motility [14]. Argov-Argaman et al. observed that impairments in the lipid composition of sperm tails during summer could result in reduced motility and velocity [15]. Cheng et al. identified 12 significant lipids, such as Lysophosphatidylserine (LPS) (20:4) and Lysophosphatidic Acid (LPA) (20:5), which influence the sperm acrosome response in humans [16]. Lu et al. proposed a link between the defective motility of sperm and lipoprotein alterations [17]. These findings underscore the critical role of lipid metabolism in seminal plasma for maintaining optimal sperm motility.

Simmental cattle, renowned for their dual-purpose traits in milk and meat production, are characterized by their rapid growth potential, high meat quality, and adaptability to various environments. Over the past two decades, they have been widely used in crossbreeding with Chinese native beef breeds to improve production efficiency [18]. However, the effect of seminal plasma lipids on sperm motility and their associated mechanisms in Simmental bulls remain unclear.

The objective of this study was to explore the relationship between sperm motility and the seminal plasma lipid profile in Simmental bulls, and to identify key lipids that potentially influence sperm motility.

## 2. Materials and Methods

### 2.1. Animals and Sample Collection

Healthy Simmental bulls (*n* = 26) with an average age of 4.9 ± 0.8 years were enrolled from a livestock breeding center in Gansu province (Wuwei, China). Semen samples were collected using the artificial vagina method [19] and were immediately transported to the laboratory for semen quality analysis within 10 min.

### 2.2. Evaluation of Sperm Quality Parameters

The volume of semen was recorded, and sperm motility, concentration, and viability were evaluated using computer-assisted sperm analysis (CASA) (MDO605, ML-300; Mailang, Foshan, China) following a previously described method [20]. CASA slides were loaded with 10 μL of each sperm sample, and a minimum of 200 sperm per sample was analyzed to determine overall motility, as recommended by the CASA software (MDO605, ML-300; Mailang, Foshan, China). Subsequently, the samples were grouped based on their motility into the high sperm motility group (HSM, >65%, N = 14) and the low sperm motility group (LSM, <65%, N = 12). Further parameters were evaluated using CASA, including straight-line velocity (VSL), curvilinear velocity (VCL), averaged path velocity (VAP), amplitude of lateral head displacement (ALH), beat-cross frequency (BCF), straightness (STR), wobble coefficient (WOB), and malformation rate. Acrosome integrity was evaluated using the fluorescein isothiocyanate-conjugated peanut agglutinin (FITC-PNA) staining method, following the protocol of Sperm Morphology FITC-PNA staining kit (cat. no. GMS14015.1.1; Genmed Scientifics, Shanghai, China), consistent with the detailed methods described by previous studies [21,22].

### 2.3. Collecting and Pretreating Seminal Plasma Samples

All semen samples were centrifuged at 3000× *g* for 10 min at 4 °C, and the supernatant was collected and stored at −80 °C. When ready for use, the seminal plasma samples were thawed at 4 °C. Each sample was vortexed for 10 s and then centrifuged at 16,000× *g* for 5 min at 4 °C. Fifty microliters of each seminal plasma sample were placed into a centrifuge tube containing 1 mL of a methanol methyl tert-butyl ether (MTBE) mixed solution. An internal standard, dimethyl sulfoxide, was added to the MTBE extract solution. After vortexing for 2 min and ultrasonication for 5 min, 500 µL of water was added to the mixture. The samples were stirred for 1 min and centrifuged at 16,000× *g* for 10 min at 4 °C. The supernatant (500 µL) was transferred to a centrifuge tube and dried under a stream of nitrogen. The resultant powder was dissolved in 100 µL of mobile phase B [acetoni-trile/isopropanol (10:90 *v*:*v*), 0.04% acetic acid, and 5 mmol/L ammonium formate] prior to being stored at −80 °C. Before liquid chromatography–electrospray ionization ion trap mass spectrometry (LC-ESI-MS/MS) analysis, quality control samples were created by mixing an equal quantity of the supernatant to assess system stability.

### 2.4. LC-ESI-MS/MS Analysis

The data acquisition instrument system consisted of ultra-performance liquid chromatography (UPLC; Shim-pack UFLC SHIMADZU CBM30A, Japan, https://www.shimadzu.com/, accessed on 20 September 2023) and tandem mass spectrometry (MS/MS; QTRAP^®^ 6500+ System, Sciex, Fremont, California, USA, https://sciex.com, accessed on 20 September 2023). The liquid phase analysis conditions were as follows: a Thermo C30 UPLC column (Thermo Scientific, Waltham, USA, 2.6 μm, 2.1 mm × 100 mm) was used. Solvent system A consisted of acetonitrile/water (60:40 *v*/*v*) with 0.04% acetic acid and 5 mmol/L ammonium formate, and solvent system B, which was acetonitrile/isopropanol (10:90 *v*/*v*) with 0.04% acetic acid and 5 mmol/L ammonium formate. The gradient program was as follows: A/B 80:20 *v*/*v* at 0 min, 50:50 *v*/*v* at 3 min, 35:65 *v*/*v* at 5 min, 25:75 *v*/*v* at 9 min, 10:90 *v*/*v* at 15.5 min; flow rate, 0.35 mL/min; temperature, 45 °C; and sample volume, 2 µL. Mass spectrometry conditions were as follows: the electrospray ionization (ESI) temperature was 550 °C, the mass spectrometry voltage was 5500 *v*, the curtain gas pressure was 35 psi, and collision-activated dissociation was set to medium. Linear ion trap (LIT) and triple quadrupole (QQQ) scans were acquired on a triple quadrupole–linear ion trap mass spectrometer (QTRAP; QTRAP^®^ 6500+ LC-MS/MS, SCIEX, MA, USA) equipped with an ESI interface. The system was controlled by Analyst^®^ software (Sciex, Framingham, MA, USA, version 1.6.3) operating in Turbo Ion Spray mode.

### 2.5. The Lipidomic Data Quality Control

The qualitative and quantitative analyses of lipids in the seminal plasma were conducted using Analyst^®^ software (version 1.6.3). After obtaining data from the samples, the extracted ion peaks of all lipids were individually integrated and corrected. The distinctive ions and signal intensities of each lipid were identified. The mass spectra of the samples were processed and corrected using MultiQuant software (version 3.0.3) (AB SCIEX, Concord, ON, Canada). The peak area was used to represent the relative abundance of each substance.

### 2.6. Statistical Analysis

The sperm quality parameters were analyzed using IBM SPSS Statistics 18.0 (IBM Corp., Armonk, NY, USA) and GraphPad Prism software (v6.0; GraphPad Software Inc., La Jolla, CA, USA). The fluorescence intensities of sperm acrosome integrity images were determined by using Image J software (version 1.8.0, National Institutes of Health, Bethesda, MD, USA), and the proportion of sperm with intact acrosome to the total sperm of individual image was calculated by Excel (version 2019, Microsoft, Redmond, WA, USA) and analyzed with an unpaired *t*-test through IBM SPSS Statistics 18.0 (IBM Corp., Armonk, NY, USA). All data are presented as the mean ± standard error of the mean (SEM), and a value of *p* < 0.05 was considered statistically significant.

Multivariate statistical analysis was conducted using the SIMCA-P program (V 14.0, Umetrics, Umea, Sweden), mainly including principal component analysis (PCA) and orthogonal correction partial least squares discriminant analysis (OPLS-DA). OPLS-DA models were verified based on the interpretation of the variation in Y (R^2^Y) and forecast ability based on the model (Q^2^). Cross-validation and permutation tests with 200 iterations showed the stability and reliability of the model at the condition of R^2^Y > 0.4 and Q^2^ > 0.4. Variable importance in a projection (VIP) value > 1 and the fold change (FC ≥ 2 or FC ≤ 0.5) in OPLS-DA, and *p* ≤ 0.05, respectively) were used as screening criteria. Significant differential lipids between the HSM and LSM groups were screened and used for metabolic pathway enrichment analysis. Kyoto Encyclopedia of Genes and Genomes (KEGG) pathway analysis was performed using MetaboAnalyst 6.0 (http://www.metaboanalyst.ca, accessed on 20 September 2024) software. A receiver operating characteristics curve (ROC) analysis was performed by Origin 8.0 software (OriginPro 2021; OriginLab Corporation, Northampton, MA, USA).

## 3. Results

### 3.1. Sperm Functional Analysis in High and Low Sperm Motility Samples

The semen quality parameters are shown in Table 1. A total of 14 samples were categorized into the HSM, while 12 samples were classified into the LSM group. There were no significant differences in semen volume or concentration between the two groups (*p* > 0.05, Table 1). The sperm motility and viability were higher in the HSM group (77.45% ± 1.86% and 87.42% ± 1.67%, respectively) compared to the LSM group (51.33% ± 4.67% and 59.81% ± 5.49%) (*p* < 0.05, Table 1).

**Figure 1:**
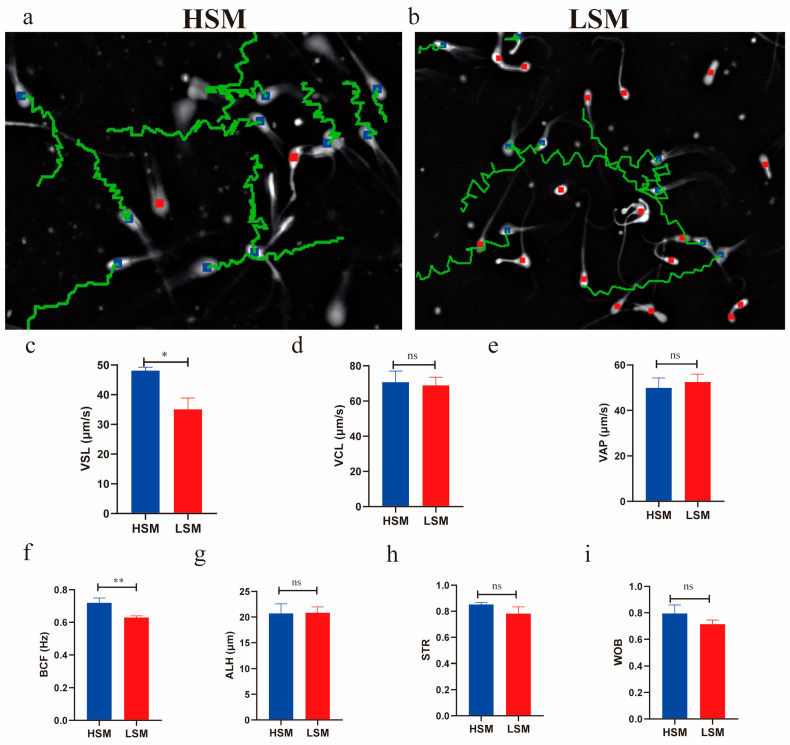
Comparison of sperm movement parameters between HSM and LSM groups. Images of sperm tracks in the HSM (**a**) and LSM (**b**) group. Red squares represent non-motile sperm, while green tracks represent motile sperm. The VSL, VCL, VAP, BCF, ALH, STR, and WOB parameters for the HSM and LSM groups are shown in panels (**c**–**i**), respectively. Data are expressed as the mean ± SEM. * and ** indicate *p* < 0.05 and *p* < 0.01, respectively, while “ns” represents no significant difference (*p* > 0.05).

The comparison of sperm movement parameters between the HSM and LSM groups is presented in Figure 1. The VSL and BCF were higher in the HSM group (48.12 ± 1.12, 0.71 ± 0.03, respectively) compared to the LSM group (35.06 ± 3.84 and 0.63 ± 0.01, respectively) (*p* < 0.05, Figure 1c,f). There were no significant differences observed in VCL, VAP, ALH, STR, or WOB between the two groups (*p* > 0.05, Figure 1d,e,g–i).

### 3.2. Differences in Sperm Malformation and Acrosome Integrity Between HSM and LSM Groups

**Figure 2:**
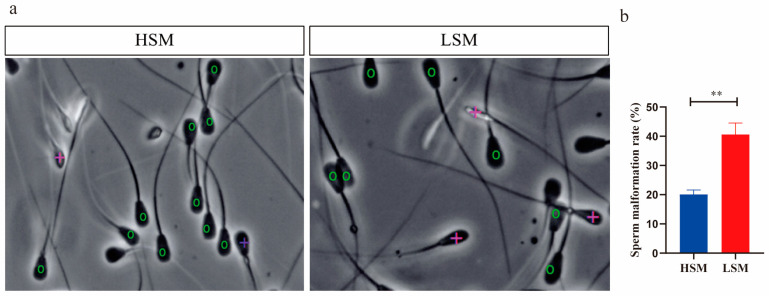
Comparison of sperm malformation between HSM and LSM groups. (**a**) Representative images of sperm morphology in the HSM and LSM groups. The “0” in green indicates normal sperm, and the “+” in purple represents the sperm with head abnormalities. (**b**) Comparison of the sperm malformation rates of the HSM and LSM groups, presented as mean ± standard deviation. Statistical significance is indicated, with ** *p* < 0.01.

**Figure 3:**
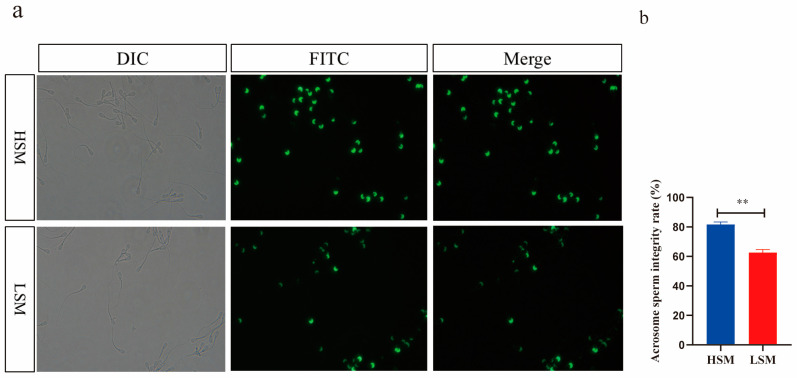
Comparative analysis of sperm acrosome integrity between the HSM and LSM groups. (**a**) Green fluorescence in the acrosomal region of the sperm head indicates intact acrosomes. (**b**) Quantitative assessment of acrosome integrity between the HSM and LSM groups, presented as mean ± standard deviation. Statistical significance is denoted by ** *p* < 0.01. The DIC image depicts the overall sperm morphology in bright field, the FITC fluorescence image represents the acrosome integrity. The merged image combines DIC and FITC images, allowing for precise correlation between the fluorescence signal and sperm morphology.

The comparison of sperm malformation rates and acrosome integrity between the HSM and LSM groups is shown in Figure 2 and Figure 3. In the HSM group, the rate of sperm malformation was decreased compared to the LSM group (40.60% ± 3.92% vs. 20.05% ± 1.56%) (*p* < 0.05, Figure 2a,b). Conversely, acrosome integrity was increased in the HSM group compared to the LSM group (81.69% ± 1.72% vs. 62.64% ± 1.96%) (*p* < 0.05, Figure 3a,b).

### 3.3. Lipid Profile Analysis Between HSM and LSM Groups

**Figure 4:**
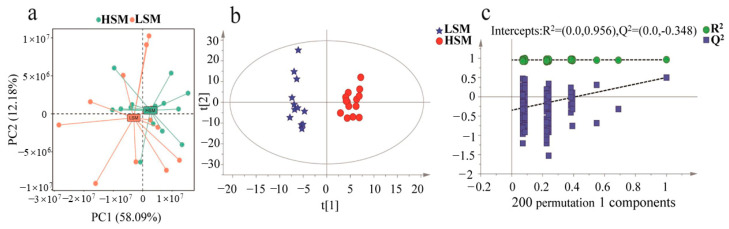
Data quality control. (**a**) Principal component analysis score plot for HSM and LSM sperm motility group samples; PC1 denotes the first principal component. PC2 denotes the second principal component. (**b**) Orthogonal partial least squares discriminant analysis score plot for the HSM and LSM group samples; t[1] = the first principal component, and t[2] = the second orthogonal component. (**c**) Permutation test plots for the HSM and LSM group samples.

The retention time and response intensity of each peak were overlaid, and the total ion chromatogram (TIC) peak shape was intact. The separation between adjacent peaks was distinct, demonstrating that the method was both reliable and stable. The PCA results revealed that the samples from both groups were within the 95% confidence interval, indicating good repeatability of the experiment (Figure 4a). The OPLS-DA analysis showed significant differences in lipid content between the HSM and LSM groups. The model parameters, R^2^Y (0.962) and Q^2^ (0.503), indicated that the model was reliable and stable (Figure 4b). Additionally, the parameters for model fitness (R^2^) and predictive ability (Q^2^) confirmed the predictability of the OPLS-DA model (Figure 4c).

### 3.4. Differential Lipids Analysis Between HSM and LSM Groups

**Figure 5:**
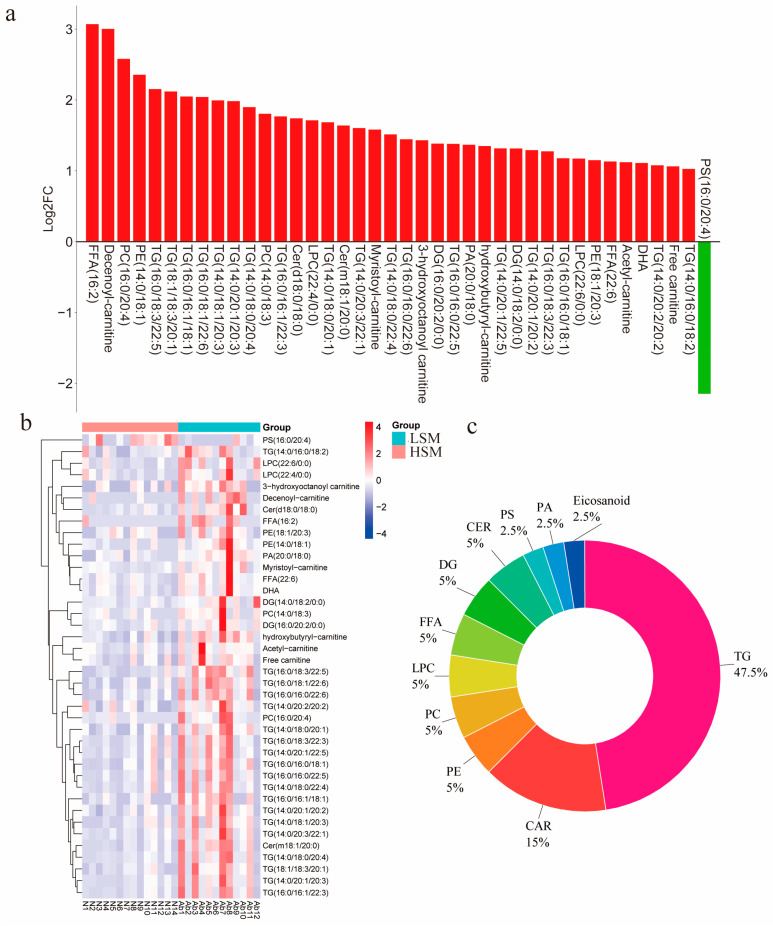
Screening and chemical classification of the differential lipids. (**a**) The bar graph represents the mean fold change values of differential lipids between HSM and LSM groups. Red indicates upregulated lipids, and green represents downregulated lipids. (**b**) Clustering heat map of the differential lipids of HSM and LSM groups. (**c**) Chemical classification of the differential lipids.

Forty differential lipids were identified in the seminal plasma between the HSM and LSM groups (Figure 5a). Triglyceride (TG), phosphatidylethanolamine (PE), phosphatidylcholine (PC), lysophosphatidylcholine (LPC), free fatty acid (FFA), carnitine (CAR), diglyceride (DG), and ceramides (CER) were all upregulated, while phosphatidylserine (PS) was downregulated in the LSM group compared to the HSM group (Figure 5a). Clustering analysis revealed that lipids enriched in the HSM group were present in lower abundance in the LSM group (Figure 5b). Further chemical classification of these differential lipids showed that the majority were TGs (47.5%) and CARs (15%), followed by PEs, PCs, LPCs, FFAs, DGs, and CERs (Figure 5c).

### 3.5. Pathway Enrichment Analysis and ROC Analysis of the Differential Lipids

**Figure 6:**
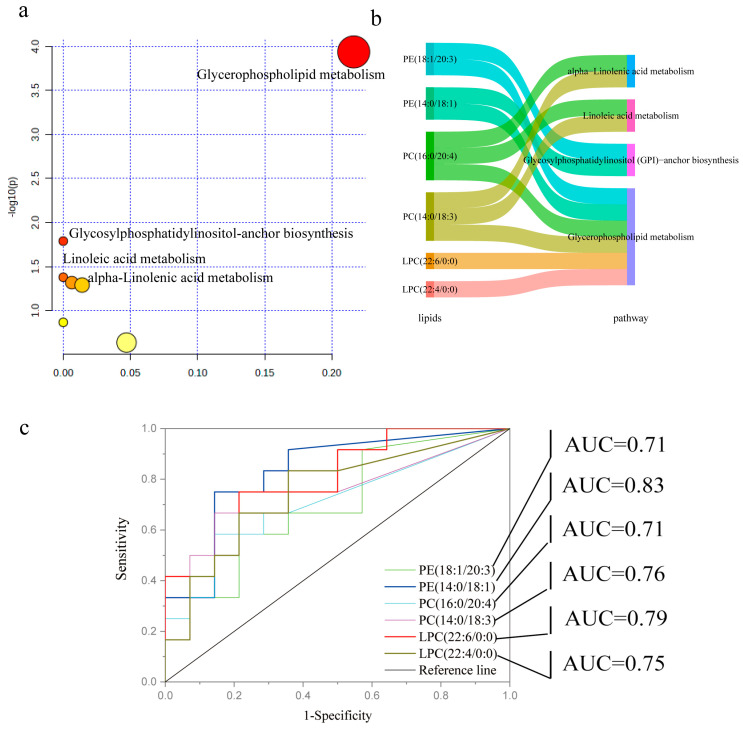
Pathway enrichment analysis and ROC analysis of the differential lipids. (**a**) Metabolic pathway analysis. The y-axis represents −log10 (*P*), and the x-axis represents the pathway impact score. The pathway impact scores are shown in circles, with darker rings indicating metabolite alterations in the relevant pathway. The dot plot illustrates the pathway impact score and the total number of metabolites in each enriched pathway (false discovery rate-adjusted *p* < 0.05). (**b**) Sankey plot showing key lipids involved in significantly enriched pathways. (**c**) ROCs for 6 key lipids. The AUC (area under ROC) is a standard metric used to evaluate the performance of a classification model. An AUC > 0.7 indicates strong predictive performance.

To further identify key lipids associated with sperm motility, metabolic pathway enrichment analysis was performed using MetaboAnalyst 6.0. Four primary metabolic pathways were significantly enriched between the HSM and LSM groups (*p* < 0.05, Figure 6a). These pathways included glycerophospholipid metabolism, glycosylphosphatidylinositol (GPI)—anchor biosynthesis, linoleic acid metabolism, and α-Linolenic acid metabolism. Six key differential lipids were involved in these pathways, including PC (16:0/20:4), PC (14:0/18:3), LPC (22:4/0:0), LPC (22:6/0:0), PE (14:0/18:1), and PE (18:1/20:3) (Figure 6b). Subsequently, receiver operating characteristic (ROC) analysis demonstrated that these six key lipids exhibited high diagnostic accuracy (AUC > 0.7, Figure 6c) and may serve as potential biomarkers for sperm motility.

## 4. Discussion

Sperm motility, viability, and velocity are critical parameters used to evaluate the potential fertility of sperm [23,24,25]. The VSL, VCL, VAP, and STR can assess sperm progression, while ALH and BCF are indicators of sperm viability [26]. In this study, the VSL and BCF values were higher in the HSM group compared to the LSM group. A higher VSL provides a competitive advantage in sperm competition [27]. Sloter et al. found that VSL values significantly decrease in 55-year-old men compared to those in 25-year-old men (50.5 vs. 45.4 μm/s) [28]. Additionally, VSL values are higher in fresh sperm compared to uncapacitated sperm [29]. Therefore, the higher VSL values observed in the HSM group indicate enhanced sperm movement capability. The BCF value indicates the frequency of sperms crossing their average movement trajectory, and a high BCF value is a good indicator of sperm movement ability [30,31]. Adequate BCF is necessary for sperm to move through the oviduct mucus and other barriers during fertilization [32]. The reduced BCF observed in the LSM group suggests a lower frequency of sperm movement along their trajectory path. Morphological abnormalities in spermatozoa are associated with reduced fertility [33,34]. Several studies have revealed a positive correlation between sperm viability and the percentage of live sperm with intact acrosomes [35,36]. In this study, the LSM group exhibited a higher percentage of sperm malformation and lower acrosome integrity compared to the HSM group. Therefore, high-motility sperm have higher VSL, BCF, and acrosome integrity, as well as a lower sperm malformation rate than low-motility sperm.

There were significant differences in lipid species abundance between HSM and LSM groups, particularly in the levels of seminal plasma triglycerides (TGs) and carnitines (CARs), based on the chemical category classification in this study. TGs serve as metabolic energy sources for sperm and act as substrates for producing glycerol in mature boar sperm [37]. Similarly, TGs are an energy source for the spermatozoa of sea urchin species [38]. However, excessive TG levels may be harmful to sperm. For example, Diaz-Fontdevila et al. found that hypertriglyceridemia decreases the acrosome reaction in rabbit sperm [39]. Mendoza et al. observed that the TG levels in men with abnormal spermatozoa were higher than those in healthy men [40]. Furthermore, studies by Ergun et al. and Furse et al. reported a negative correlation between TG levels and sperm motility [41,42]. In this study, the LSM group exhibited higher TG concentrations in seminal plasma compared to the HSM group, consistent with previous findings. The excessive accumulation of TGs that cannot be utilized in time may lead to lipotoxicity, impairing sperm function [42]. Therefore, elevated TG levels in seminal plasma may negatively impact sperm motility.

**Figure 7:**
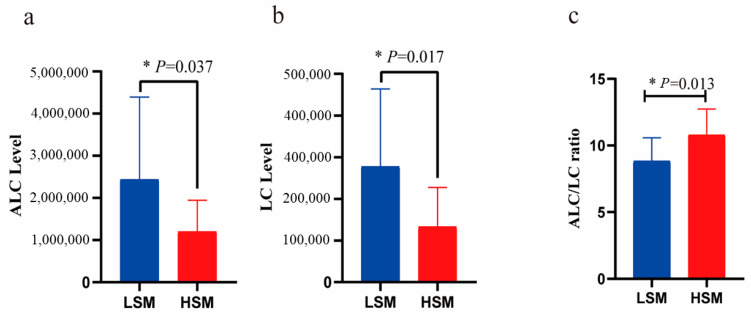
The levels of ALC (**a**) and LC (**b**), and ALC: LC ratio (**c**) between HSM and LSM groups.

CARs are essential carriers for sperm motility and capacitation, providing readily available energy for spermatozoa [43]. This beneficial effect is mediated by the transport of long-chain fatty acids toward or across the inner mitochondrial membrane, facilitating β-oxidation [44]. Supplementation with L-carnitine (LC) during the liquid storage of boar semen has shown no significant beneficial effects on the maintenance of sperm motility [45]. Moreover, LC has been reported to inhibit sperm progressive motility in bovine and rooster sperm by reducing oxygen consumption in spermatozoa [46,47]. The epididymal tissue contains high levels of LC and acetyl-carnitine (ALC) [48], and evidence has demonstrated a positive correlation between the ALC/LC ratio and sperm motility [43]. Bartellini et al. found that samples characterized by asthenozoospermia exhibited higher LC and ALC levels but a significantly reduced ALC/LC ratio [49]. In this study, compared to the HSM group, the concentrations of ALC and LC were elevated, while the ALC/LC ratio was reduced in the LSM group (Figure 7). This finding aligns with previous studies [49]. The reduction in the ALC/LC ratio could be explained by an impairment of the enzymatic system (carnitine transferase) that controls the reaction between LC and ALC, leading to defective spermatozoa motility [43]. Consequently, a decreased ALC/LC ratio in seminal plasma negatively impacts sperm motility.

Lipids that showed significant changes in glycerophospholipid metabolism included PC, LPC, and PE. LPC has been found to impact the acrosome reaction in spermatozoa by damaging the sperm membrane [50]. Additionally, the level of LPC in seminal plasma has been shown to have a negative correlation with sperm motility [51]. Similarly, LPC (22:6) has been identified as a reliable marker of spermatozoa lipid oxidation [52]. LPC is an inverted cone-shaped phospholipid that can form both curved bilayers and non-bilayers, potentially leading to unstable membrane domains [53]. These findings indicated that elevated LPC levels in seminal plasma impair sperm motility by disrupting membrane structure.

In addition, PCs and PEs are the most abundant phospholipids in mammalian cell membranes and participate in regulating oxidative stress by serving as methyl group donors [54]. PCs are converted into PSs under the catalysis of phosphatidylserine synthase and subsequently converted into PEs by phosphatidylserine decarboxylase [55]. The increased levels of PCs in chicken sperm have been reported to be negatively associated with fertility during aging [56]. A study revealed that lower levels of PEs may contribute to the stabilization of the bilayer structure in cellular and acrosomal membranes [51]. In this study, the levels of PE (18:1/20:3), PE (14:0/18:1), PC (16:0/20:4), and PC (14:0/18:3) were increased in the LSM group compared to the HSM group. Consequently, we established a negative correlation between sperm motility and elevated levels of LPCs, PCs, and PEs in the seminal plasma.

## 5. Conclusions

This study demonstrated that high-motility sperm has higher VSL, BCF, and acrosome integrity, as well as a lower sperm malformation rate, compared to low-motility sperm. The elevation of 39 differential lipids in seminal plasma, primarily enriched in glycerophospholipid and linoleic acid metabolism, was associated with a negative impact on sperm motility. Specifically, PC (16:0/20:4), PC (14:0/18:3), LPC (22:4/0:0), LPC (22:6/0:0), PE (14:0/18:1), and PE (18:1/20:3) appeared to be applicable as biomarkers of sperm motility. This study established correlations between sperm motility in bulls and lipid metabolism in seminal plasma, providing a reference for the evaluation of fertility in Simmental bulls.

## Figures and Tables

**Table 1:** Quality parameters of HSM and LSM group.

	Groups		
Parameters	HSM	LSM	*p*-Value
Sperm collection/mL	5.90 ± 1.47	6.78 ± 2.58	0.32
Sperm concentration (×10^8^)	1.06 ± 4.70	1.05 ± 3.99	0.31
Sperm motility (%)	77.45 ± 1.86 ^A^	51.33 ± 4.67 ^B^	<0.01
Sperm viability (%)	87.42 ± 1.67 ^A^	59.81 ± 5.49 ^B^	<0.01

Note: Comparison of sperm quality parameters between the HSM and LSM groups. Data are expressed as mean ± SEM. The absence of letters in the same row indicates no significant difference between treatments (*p* > 0.05); (A/B) in the same row indicate a significant difference (*p* < 0.01).

## Data Availability

The dataset is available upon request from the authors.
